# Cancer-related changes and low-to-moderate exposure to welding fumes: A longitudinal study

**DOI:** 10.5271/sjweh.3988

**Published:** 2021-12-30

**Authors:** Ulrike Maria Dauter, Ayman Alhamdow, Andrea Cediel-Ulloa, Anda Roxana Gliga, Maria Albin, Karin Broberg

**Affiliations:** 1 Institute of Environmental Medicine, Karolinska Institute, Stockholm, Sweden; 2 Department of Organismal Biology, Uppsala University, Uppsala, Sweden; 3 Division of Occupational and Environmental Medicine, Department of Laboratory Medicine, Lund University, Lund, Sweden

**Keywords:** AHRR, B3GNTL1, DNA Methylation, F2RL3, lung cancer, mitochondrial DNA, occupational exposure, respirable dust, telomere length

## Abstract

**Objective:**

This study tested for an association between early cancer-related biomarkers and low-to-moderate exposure to fumes from welding mild steel.

**Methods:**

Male, non-smoking participants from southern Sweden were recruited and examined (N=338, 171 welders and 167 controls); of these, 78 welders and 96 controls were examined on two occasions six years apart. Exposure to welding fumes was evaluated by measuring respirable dust, welding years, and cumulative exposure. DNA methylation of CpG sites within the cancer-related genes *AHRR*, *F2RL3*, and *B3GNTL1* was measured by pyrosequencing and relative mitochondrial DNA copy number and telomere length were measured by qPCR in whole-blood samples. Multivariate models were used for longitudinal analysis.

**Results:**

Median exposure to respirable dust was 0.7 mg/m^3^ at both timepoints, adjusted for use of personal protective equipment. Compared with controls, welders showed a significant decrease over time in DNA methylation of *B3GNTL1* CpG1 and CpG4 [adjusted for age, body mass index, and smoking: β=-0.66, standard error (SE)=0.28; β=-0.48, SE=0.24, respectively]. In addition, exposure to respirable dust and cumulative exposure was associated with a decrease in methylation of *F2RL3* CpG2 among all welders (adjusted β=-0.67, SE=0.23 and β=-0.03, SE=0.02, respectively). No significant associations were found for *AHRR*, mitochondrial DNA copy number, or telomere length.

**Conclusion:**

Low-to-moderate exposure to welding fumes was associated with a small effect on selected early epigenetic biomarkers of cancer. The direction of the methylation pattern (lower methylation of specific CpG sites) indicates early lung cancer-related changes associated with mild steel welding.

Welders are exposed to high levels of welding particles (fine and ultrafine), gases, and ultraviolet radiation, and in some cases, co-exposure to asbestos and solvents may take place (1). In 2017, the International Agency for Research on Cancer (IARC) classified welding fumes as “carcinogenic to humans” (Group 1) (1, 2). This classification was based on epidemiological studies showing an increased lung cancer risk in welders and is valid for both mild and stainless steel welding (2). The main carcinogenic components of welding fumes are considered to be respirable particles that are 20–1000 nm in size and consist of a mixture of different metals, such as iron, manganese, chromium, and nickel. However, the composition of welding fumes varies, depending on the type of electrode used (mild steel or stainless steel) as well as the type of welding process (eg, gas or arc welding) and coating of the metals.

Protection of workers from adverse effects of welding fumes, including setting relevant occupational exposure limits (OEL), remains an important concern for public health. Worldwide, approximately 11 million people work as welders and an additional 110 million are exposed to welding particles at work (1). In Sweden, 13 000 people work as welders (3) and >250 000 are exposed to welding fumes at work (4). The Swedish occupational exposure limit (OEL) is 2.5 mg/m^3^ for inorganic respirable dust (5), however, this OEL is not health-based. Thus, it is also not clear if this limit is sufficiently protective with regard to cancer risk. In a cohort study from 2008, an increased risk for lung cancer was observed among ’ever welders’ [standardized incident ratio: 1.35, 95% confidence interval (CI) 1.06–1.70], with suggested dose–response associations with duration of welding as well as cumulative exposure (6). Moreover, a cohort study and two case–control studies found associations between occupational welding and lung cancer (7–9).

The mechanisms underlying the carcinogenicity of welding fumes are not fully understood and numerous mechanisms have been suggested. Studies have reported systematic inflammation (10, 11), oxidative stress (12, 13), and immune suppression (14) among welders following exposure to welding fumes. Previously, our cross-sectional studies of low-to-moderately exposed Swedish welders found only limited evidence of inflammation and mild increased oxidative stress measured as 8-hydroxydeoxyguanosine in urine (15, 16). We did, however, find changes in cancer-related biomarkers: shorter telomeres (16), an increase in mitochondrial DNA copy number (mtDNAcn) among welders compared to controls (17). A more recent study observed associations with cancer-related proteins (18).

Telomeres, the repetitive DNA sequence (TTAGGG) at the end of the chromosomes, help maintain DNA integrity (19). During mitosis, telomeres shorten, and the telomere length (TL) limits how many times a cell can divide. Lifestyle factors and occupational and environmental stressors can accelerate telomere shortening or induce increases in TL. Shorter telomeres can result in chromosomal aberrations (20), a key change in carcinogenesis, whereas longer telomeres may result in higher proliferative potential and accumulation of mutations (21). Earlier case–control studies of lung cancer have shown shorter telomeres among lung cancer cases (22, 23), but more recent case–control and prospective studies revealed longer telomeres in association with lung cancer, especially adenocarcinomas (24–28).

The mtDNAcn provides another biomarker for early cancer-associated changes. The mitochondrial DNA (mtDNA) lacks introns and histones, and has a limited capacity for DNA repair, making it vulnerable to oxidative DNA damage (29). A prospective cohort study of male smokers from Southwest Finland suggested that mtDNAcn increases in cancer patients compensating for the low mtDNA functionality (30).

Changes in DNA methylation of CpG sites of cancer­related genes play a major role in cancer and are specific for some carcinogens (31). Tobacco smoking has repeatedly been associated with hypomethylation of specific CpG sites in genes such as *AHRR* (encoding aryl-hydrocarbon receptor repressor) and *F2RL3* (encoding F2R like thrombin/trypsin receptor 3) (32) in peripheral blood cells. Hypomethylation of cg03636183 in *F2RL3* (referred to as *F2RL3* CpG2 in the current study) predicts lung cancer risk (33–35). Our previous cross-sectional study of non-smoking welders showed that *F2RL3* CpG2 hypomethylation was associated with working as a welder, previous smoking, and exposure to respirable dust (36). Additional evidence shows that lower CpG cg05575921 methylation in *AHRR* (referred to as: *AHRR* CpG3 in the current study) predicts lung cancer and lymphoblastic leukemia (34). Hypomethylation of *PC* (pyruvate carboxylase; CpG cg10151248) and *B3GNTL1* (beta1,3-N-acetylglucosaminyltransferase-like protein 1; CpG cg13482620; referred to as *B3GNTL1* CpG6 in the current study), were also shown to be associated with lung cancer development independent of smoking in a recent cohort study (37). Therefore, exploration of epigenetic changes provides useful information to assess cancer risk.

The aim of this study was to evaluate early cancer-related changes, including TL, mtDNAcn, and DNA methylation of selected genes, in a cohort of Swedish welders exposed to low-to-moderate levels of welding fumes and measured six years apart.

## Methods

### Study design

A cohort of male welders and non-exposed controls from southern Sweden (Södra sjukvårdregionen) was established in 2010 (15). Baseline examination (timepoint 1) included 101 welders working in small- and medium-sized welding companies, and 127 age-matched controls working as gardeners and janitors for a municipality or as workers in food-storage facilities. The control group had very low or no occupational exposure to particles, including welding fumes (15). Inclusion criteria were being a non-smoker for at least the previous six months and being male. Follow-up was conducted six years after the baseline recruitment (years 2016/2017, timepoint 2). The drop-out rate was 23% (N=23) among welders and 24% (N=31) among controls; mainly due to retirement and closing of one of the companies. During follow-up additionally 70 welders and 40 controls were recruited. Based on the questionnaire data a few current smokers, who had been nonsmokers at baseline, were identified at timepoint 2 (2 welders, 3 controls).

In total, the study cohort included 338 individuals: 171 welders and 167 controls. Of these 338 individuals, 142 (78 welders, 96 controls) were examined at both timepoint 1 and timepoint 2, whereas 164 were examined only at timepoint 1 or timepoint 2 (93 welders, 71 controls).

All participants were asked to complete a questionnaire, including questions about country of birth, education, personal and family history of cancer, diet, physical activity, smoking history, use of snus (Swedish moist tobacco), alcohol, current residency, as well as exposure to particle/ smoke during leisure time.

Peripheral blood from welders and controls was collected at both timepoints by the same nurse.

### Exposure assessment

A structured questionnaire was used for controls and welders to gain information about their occupational history, including their present and past workplaces, type and duration of jobs, and whether they were exposed to welding or diesel fumes. Additionally, welders were asked about the type of welding they performed at work, how many hours they spent welding on average per work week, their individual work station, use of area-level or point-source exhaust, as well as their use of personal respiratory, noise, and eye protection devices while welding.

### Personal respirable dust measurements

Personal sampling of respirable dust was performed for the welders and area-level dust monitoring was performed for the controls. A detailed description can be found in previous publications (16, 18, 38). Based on measurements from timepoint 2, the major elements in the welding fumes were iron and manganese (iron median exposure 0.5515 mg/m^3^; manganese 0.0896 mg/m^3^), whereas exposure to chromium and nickel was at much lower levels (chromium median exposure 0.0004 mg/m^3^; nickel 0.0005 mg/m^3^) as described in an earlier paper (39).

The measured respirable dust concentrations were corrected for use of protective devices by a correction factor of 3, as described in earlier papers (16, 18, 38). At timepoint 2, five welders had new or different personal protective devices compared to at timepoint 1. One welder upgraded to a half-mask (correction factor of 2), whereas four welders had a new version of a powered air purifying respirator with a double visor (correction factor of 50).

To determine the respirable dust, the filtered samples were gravimetrically analyzed according to a validated method (40). The limit of detection was 0.05 mg/sample.

For welders with missing exposure data, we based their exposure on data from welders working in the same company and with the same work tasks.

At timepoint 1, 53 out of 101 welders had measured respirable dust concentrations and 48 had estimated concentrations. At timepoint 2, 103 welders had measured respirable dust concentrations, 20 had estimated concentrations, and no data was available for 22 welders. Finally, 22 welders had measured respirable dust concentrations for both timepoints 1 and 2.

Detailed information about area level dust monitoring in the control companies have been published previously (15, 18).

To reflect the actual exposure, respiratory dust concentrations adjusted for personal respiratory protection were used in the calculation of the cumulative exposure and the statistical analysis.

### Cumulative exposure

The cumulative exposure (or cumulative dose) for timepoint 1 was estimated by multiplying the respirable dust data (adjusted by use of personal protection devices) and the reported years spent welding (18). Similar calculations were made for timepoint 2, adding the estimate from timepoint 1.

### Telomere length, mtDNA copy number, and DNA methylation

DNA extraction from peripheral blood samples of welders and controls was done using the QIAmp DNA Blood Midi kit (Qiagen, Hilden, Germany). Relative TL was measured using quantitative PCR (qPCR) (LightCycler 480, Roche, Basel, Switzerland) applying a SYBR Green-based assay established by Cawthon (41) as previously described (42, 43) with minor adjustments.

Similarly, the relative mtDNAcn was measured as the M/S ratio, with M as the mtDNAcn and S as the single-copy gene *HBB*.

Eleven CpG sites were investigated: three in *AHRR* (CpG1–CpG3 [CpG3 corresponds to cg05575921 Illumina 450K]), two in *F2RL3* (CpG1 and CpG2 [corresponds to cg03636183]), and six in *B3GNTL1* (CpG1–CpG6 [CpG6 corresponds to cg13482620]). Supplementary Table 1 provides detailed information about the genomic locations of all CpG sites.

More detailed information can be found in the supplementary material (https://www.sjweh.fi/article/3988) under methods.

### Statistical analysis

The continuous variables are presented as median and 5–95^th^ percentiles whereas categorical variables are presented as frequencies and percentages based on the total valid answers from the questionnaires. Evaluation of differences between exposure groups (welders and controls) were done with the Kruskal-Wallis rank-sum test followed by Dunn’s post hoc test when comparing three groups or more; Wilcoxon Unpaired Two-Sample test was used for continuous variables when comparing two groups, and Fisher’s exact test was used for categorical variables.

A detailed method description regarding differences in epigenetic biomarkers between never and ever smokers, differences between recruitment groups and the relationship between smoking and the outcome and exposure variables can be found in the supplementary material.

The correlation between selected variables of interest was analyzed using the R package *corrplot*. Spearman correlation was used, and the data were ordered by hierarchical clustering. The correlation coefficient was determined by the base R function *cor.test*.

Longitudinal analysis employing linear mixed models was used to evaluate associations between exposure groups and DNA biomarkers (CpG sites, mtDNAcn, TL), and fitted by using the *lmer* function from the *lme4* package in R. Participants were included as random factors (random intercepts) in the mixed model, whereas age, body mass index (BMI), smoking (if the participants ever smoked in their life) and group were included as fixed factors. The *RsgGLM* function from the R package *MuMin* was used to calculate the explained variance by fixed factors (*R*^2^_m_) and random factors (*R*^2^_c_). *P*-values were adjusted for multiple testing using the Benjamini-Hochberg method. Sensitivity analysis was performed including (i) only controls who never welded and (ii) only never-smokers.

Mixed models were used for evaluating associations between measurements of exposure (separate analyses for welding years, respirable dust in mg/m^3^ and cumulative exposure in years) and DNA biomarkers, where welders were included as random factors (random intercepts), and age, BMI, smoking (ever smoked) were included as fixed factors. Sensitivity analysis included (i) only welders with measured respirable dust data for at least one timepoint and (ii) only never-smokers.

All statistical analysis was performed in R 3.6.1 (44).

### Ethical approval

The study was done in accordance with the 1964 Helsinki Declaration. All study participants gave their informed consent to take part in the study, and the Regional Ethical Committee of Lund University, Sweden, approved the study (2010/132).

## Results

### Characteristics of the study participants

Table 1 details the demographics and lifestyle factors of the study participants. No significant differences between welders and controls were observed in age, BMI, or smoking status, and both groups had relatively healthy lifestyles, with low alcohol consumption, regular exercise, a balanced diet, and little tobacco consumption, apart from a few ‘party smokers’ (table 1). No difference was observed for respirable dust levels between timepoint 1 and 2. Increases in physical activity and cancer history were found at timepoint 2 in the welding group. The respirable dust concentrations adjusted for use of personal protective equipment (PPE) showed a median of 0.7 mg/m^3^ at both timepoints, but there was a wider range at timepoint 1 compared to timepoint 2.

**Table 1A:** Characteristics of welders and controls divided by timepoint.

Continuous variables	Timepoint 1		Timepoint 2		P-value welders ^a^	P-value controls ^a^
	
	Welders N=101	Controls N=127	Welders N=145	Controls N=134		
			
	Median (5–95^th^ percentile)	Median (5–95^th^ percentile)	Median (5–95^th^ percentile)	Median (5–95^th^ percentile)		
Age (years)	41 (23–60)	43 (23–56)	47 (27–64.8)	48 (29–62)		
Welding years	7 (1–24)	0 (0–11.7)	11 (2–30)	0 (0–9)	0.002	
Respirable dust (mg/m^3^) ^b^	1.3 (0.2–4.2)		1.24 (0.1–5.5)		0.673	
Respirable dust adjusted (mg/m^3^) ^c^	0.7 (0.2–4.2)		0.7 (0.1–2.4)		0.353	
Cumulative exposure ^d^	4.6 (0.4–46.7)		8.44 (0.8–35.8)		0.060	
Body-mass index (kg/m^2^)	27.7 (21.8–34.4)	27.1 (22.4–33.9)	28.5 (22.6–37.1)	27.8 (22.2–34.7)	0.168	0.160

**Table 1B:** Characteristics of welders and controls divided by timepoint.

Categorical variables ^e^	Timepoint 1		Timepoint 2		P-value welders ^a^	P-value controls ^a^
	
	Welders N=101	Controls N=127	Welders N=145	Controls N=134		
			
	N (% relative to the total valid answers)	N (% relative to the total valid answers)	N (% relative to the total valid answers)	N (% relative to the total valid answers)		
Country of birth (Sweden)	74 (73)	119 (94)	99 (69)	122 (92)		
Education (university or higher)	4 (4)	17 (13)	10 (7)	18 (14)	0.197 ^i^	0.532 ^i^
Residence (large and small cities) ^f^	26 (26)	57 (45)	23 (16)	63 (47)	0.074 ^j^	0.710 ^j^
Exposure to particles from a hobby ^g^	29 (29)	20 (16)	24 (17)	25 (19)	0.370	0.518
Smoking history (ever smoked)	47 (47)	47 (37)	63 (44)	50 (38)	0.697	0.609
Smoking status (current)						
Non-smoker	96 (95)	124 (98)	135 (94)	128 (96)	0.642	0.505
Party smoker	5 (5)	3 (2)	6 (4)	3 (2)		
Smoker	0 (0)	0 (0)	3 (2)	2 (2)		
Current snus use	28 (28)	24 (19)	45 (31)	28 (21)	0.554	0.666
Alcohol intake (3+ times/week)	2 (2)	4 (2)	3 (2)	5(4)	0.790 ^k^	0.919 ^k^
Vegetable intake (5+ times/week)	59 (58)	82 (65)	83 (58)	95 (71)	0.733 ^l^	0.685 ^l^
Physical activity (moderate/high) ^h^	39 (39)	53 (42)	69 (48)	64 (48)	0.046 ^m^	0.767 ^m^
Cancer history	0 (0)	2 (1)	7 (7)	2 (2)	0.025	0.963
Family cancer history	16 (17)	26 (20)	32 (22)	32 (24)	0.520	0.698

a P- values to showcase the differences at timepoint 1 and timepoint 2, Wilcoxon Unpaired Two-Sample test was used for calculation of the continuous variables, Fisher’s exact test for categorial variables.

^b^ Estimated or measured by personal sampling.

^c^ Adjusted values considering the use of personal respiratory protection equipment.

^d^ Calculated values based on adjusted respirable dust data and years spent welding.

e Categorization refers to “yes” or “no” unless stated otherwise.

f Towns and countryside compared to small and large cities.

g Particle exposure during leisure time activities, including welding fumes, dust, engine exhaust or engine diesel.

h Physical activity for a minimum of 30 minutes per week that involves sweating.

i Statistical test based on five different categories of education ranging from secondary school to university studies.

j Statistical test for four different categories of current residence ranging from large city to country side.

k Statistical test for six categories of alcohol intake, ranging from every day to never.

l Statistical test based on eight categories ranging from three or more per day to never.

m Statistical test based on four categories ranging from sedentary to intensive physical activity.

Table 2 provides information about the DNA methylation, TL, and mtDNAcn at the different timepoints. Significant differences in methylation status between never- and ever-smokers were found for five sites, all of which had higher methylation in never-smokers: *AHRR* CpG1 (4.6%), *AHRR* CpG2 (4.8%), *AHRR* CpG3 (5.6%), *F2RL3* CpG1 (2.0%), and *F2RL3* CpG2 (2.1%), as well as for mtDNAcn (0.5%) (supplementary table S2).

**Table 2:** Median methylation status of the selected CpG sites of the genes in welders and controls including the 5^th^ and 95^th^ percentile as well as the median mitochondrial DNA copy number and median telomere length for timepoint 1 and timepoint 2 for both groups.

	Timepoint 1 (2010–2011)		Timepoint 2 (2016–2017)	
	
	Welders N=101	Controls N=127	Welders N=145	Controls N=134
			
	Median (5–95^th^ percentile)	Median (5–95^th^ percentile)	Median (5–95^th^ percentile)	Median (5–95^th^ percentile)
AHRR				
CpG1	75.0 (60.9–82.5)	75.6 (60.6–83.6)	74.6 (56.6–84.7)	75.4 (60.6–83.8)
CpG2	68.0 (55.9–73.2)	68.1 (54.0–74.0)	67.4 (53.9–75.0)	67.9 (53.1–73.9)
CpG3	87.8 (73.8–94.7)	88.3 (75.8–94.4)	87.1 (74.3–94.4)	88.1 (73.8–94.1)
F2RL3				
CpG1	72.1 (66.7–75.5)	72.6 (67.3–75.2)	72.0 (65.8–75.9)	71.6 (66.4–75.1)
CpG2	92.6 (85.3–100.0)	91.5 (85.1–98.7)	92.3 (87.0–100.0)	93.0 (84.1–99.5)
B3GNTL1				
CpG1	98.2 (93.0–100.0)	98.9 (95.1–100.0)	98.3 (93.3–100.0)	98.5 (93.9–100.0)
CpG2	96.9 (95.6–97.8)	96.9 (95.7–97.8)	97.0 (96.1–97.8)	96.9 (95.6–97.7)
CpG3	96.2 (91.7–100.0)	95.1 (90.6–100.0)	95.7 (90.5–100.0)	95.8 (91.5–100.0)
CpG4	95.8 (91.4–100.0)	97.0 (91.9–100.0)	96.0 (91.8–100.0)	96.4 (91.5–100.0)
CpG5	98.2 (95.1–100.0)	98.0 (94.8–100.0)	98.6 (95.3–100.0)	98.1 (95.3–100.0)
CpG6	95.8 (91.5–99.1)	95.9 (92.8–99.5)	96.1 (92.5–99.4)	96.0 (91.7–99.4)
mtDNA ^a^	1.1 (0.8–1.5)	1.1 (0.8–1.5)	1.1 (0.8–1.6)	1.2 (0.8–1.7)
Telomere length	0.9 (0.7–1.2)	0.9 (0.7–1.2)	1.0 (0.7–1.3)	0.9 (9.7–1.2)

a Mitochondrial DNA copy number.

No significant difference in characteristics was found when comparing dropouts at timepoint 1 with the new recruits at timepoint 2, or between dropouts at timepoint 1 and the remaining individuals. Use of snus differed between new recruits and cohort welders in timepoint 2, otherwise no differences were found (supplementary table S3).

To evaluate their relationships at each timepoint, we plotted lifestyle factors, exposure measures, and DNA biomarkers in a correlation heatmap (supplementary figure S1a and b). Age and BMI were significantly correlated with TL (age: P<0.001, r_S_=-0.32 at timepoint 1 and -0.31 at timepoint 2; BMI: r_S_=-0.19 at timepoint 1 and -0.17 at timepoint 2). The mtDNAcn was significantly positively correlated with TL (P<0.001, r_S_=0.25 at timepoint 1 and 0.32 at timepoint 2). Smoking status (ever- or never-smoker) was significantly correlated with differences in methylation of *AHRR* CpG1–3, and *F2RL3* CpG1–2 (*P*<0.001) (supplementary table S2). Based on these significant correlations, age, BMI and smoking status were selected as covariates for the linear mixed model analysis.

### Telomere length and mtDNAcn and welding

No significant differences in TL or mtDNA were observed between welders and controls or when examining different exposure measures among the welders over time (table 3, table 4).

**Table 3:** Association of (epi)genetic markers/sites in welders and controls using linear mixed modelling for analysis. The models are adjusted for age, body mass index, and smoking (ever smoking).

Exposed vs Unexposed	R^2^_m_ (%)	β (SE)	P-value	P-value adjusted ^a^	N ^b^
AHRR					
CpG1	2	-0.78 (0.89)	0.382	0.608	504
CpG2	1	-0.56 (0.76)	0.457	0.776	504
CpG3	3	-0.46 (0.80)	0.565	0.916	504
F2RL3					
CpG1	5	-0.01 (0.31)	0.985	0.575	504
CpG2	1	0.62 (0.44)	0.160	0.097	504
B3GNTL1					
CpG1	1	-0.66 (0.28)	0.016	0.034	503
CpG2	2	-0.01 (0.06)	0.912	0.955	503
CpG3	0	0.03 (0.26)	0.904	0.919	503
CpG4	0	-0.48 (0.24)	0.046	0.144	503
CpG5	2	0.45 (0.16)	0.006	0.034	501
CpG6	1	0.00 (0.19)	0.989	0.936	501
mtDNA ^c^	1	-0.03 (0.03)	0.231	0.515	504
Telomere length	4	0.01 (0.02)	0.503	0.638	504

a Benjamini-Hochberg adjustment.

b Number of observations.

c Mitochondrial DNA copy number

**Table 4:** Association of the epigenetic markers/sites among welders with the exposure variable expressed as respirable dust (adjusted for use of personal protective equipment), cumulative exposure, and years spend welding, using linear mixed modelling for analysis. The models are adjusted for age, body mass index, and smoking (ever smoking).

	R^2^_m_ (%)	β (SE)	P-value	P-value adjusted ^a^	N ^b^
Respirable dust					
AHRR					
CpG1	1	0.25 (0.38)	0.505	0.574	223
CpG2	0	-0.20 (0.30)	0.504	0.972	223
CpG3	6	-0.12 (0.38)	0.754	0.817	223
F2RL3					
CpG1	6	-0.40 (0.16)	0.015	0.049	223
CpG2	5	-0.67 (0.23)	0.004	0.016	223
B3GNTL1					
CpG1	7	0.28 (0.11)	0.016	0.063	223
CpG2	7	0.04 (0.04)	0.227	0.374	223
CpG3	0	0.00 (0.15)	0.987	0.968	223
CpG4	1	0.06 (0.15)	0.705	0.716	223
CpG5	1	-0.04 (0.09)	0.677	0.879	221
CpG6	2	0.02 (0.12)	0.876	0.962	221
mtDNA ^c^	1	0.01 (0.01)	0.681	0.792	222
Telomere length	3	0.00 (0.01)	0.649	0.802	222
Cumulative exposure					
AHRR					
CpG1	2	-0.03 (0.03)	0.275	0.561	222
CpG2	1	-0.03 (0.03)	0.231	0.522	222
CpG3	6	-0.02 (0.03)	0.431	0.500	222
F2RL3					
CpG1	10	-0.04 (0.01)	<0.001	<0.001	222
CpG2	3	-0.03 (0.02)	0.048	0.105	222
B3GNTL1					
CpG1	7	0.02 (0.01)	0.011	0.017	222
CpG2	6	0.00 (0.00)	0.511	0.544	222
CpG3	0	0.00 (0.01)	0.977	0.977	222
CpG4	2	0.01 (0.01)	0.504	0.689	222
CpG5	1	0.00 (0.01)	0.512	0.698	220
CpG6	2	0.00 (0.01)	0.682	0.702	220
mtDNA ^c^	1	0.00 (0.00)	0.615	0.861	221
Telomere length	3	0.00 (0.00)	0.510	0.637	221
Welding years					
AHRR					
CpG1	1	0.02 (0.08)	0.776	0.874	245
CpG2	0	0.02 (0.07)	0.807	0.955	245
CpG3	4	0.07 (0.08)	0.336	0.428	245
F2RL3					
CpG1	4	-0.02 (0.03)	0.484	0.532	245
CpG2	1	0.01 (0.04)	0.746	0.909	245
B3GNTL1					
CpG1	3	0.06 (0.03)	0.080	0.088	245
CpG2	6	-0.01 (0.01)	0.315	0.636	245
CpG3	0	0.00 (0.03)	0.893	0.975	245
CpG4	1	-0.01 (0.02)	0.647	0.803	245
CpG5	1	0.02 (0.02)	0.324	0.569	243
CpG6	2	-0.03 (0.02)	0.160	0.273	243
mtDNA ^c^	1	0.00 (0.00)	0.587	0.698	244
Telomere length	3	0.00 (0.00)	0.389	0.527	244

a Benjamini-Hochberg adjustment.

b Number of observations.

c Mitochondrial DNA copy number.

### DNA methylation and welding

Welders showed significantly lower methylation of *B3GNTL1* CpG1 (P=0.016, linear mixed model analysis, adjusted for age, BMI, and ever smoking) and CpG4 (P=0.046) (table 3) compared with controls. No significant differences between exposure groups were found for *AHRR* or *F2RL3*, but effect estimates for *AHRR* were in general negative: all CpG sites showed lower methylation in welders compared with controls. Welders also showed an increase of methylation of *B3GNTL1* CpG5 (P=0.006) compared with controls. The associations were still significant for *B3GNTL1* CpG1 and CpG5 after adjusting for multiple testing.

Sensitivity analysis including (i) only controls who had never welded, showed a decrease of the effect estimates of *B3GNTL1* CpG1, which became nonsignificant (P=0.076, β=-0.54, SE=0.30) whereas the effect increased for CpG4 and remained significant (P=0.11, β=-0.66, SE=0.26). Sensitivity analysis including (ii) only individuals who had never smoked, showed similar directions of effect estimates as the main analysis (supplementary table S4). The effect estimates increased from the main analysis for *B3GNTL1* CpG1 and CpG4 and remained significant (CpG1 β=-1, SE=0.41 and CpG4 β=-0.69, SE=0.33). Welders also showed a significant increase in methylation of *B3GNTL1* CpG5 in both sensitivity analyses: (i) P=0.012, β=0.42, SE=0.17, (ii) P=0.014, β=0.56, SE=0.23 (supplementary table S3).

### Dose–response relationships with DNA methylation among welders

Respirable dust (adjusted for PPE) and cumulative exposure showed the strongest associations with DNA methylation in the welders (N=220–223) (table 4). A significant decrease in methylation was observed for *F2RL3* CpG1 (P=0.015) and CpG2 (P=0.004) with increasing respirable dust. Cumulative exposure was also associated with lower methylation of *F2RL3* CpG1 (P=<0.001) and CpG2 (P=0.048). No associations were found with welding years (N=243–245). Significance remained after adjustment for multiple testing for *F2RL3* CpG1 in associations with respirable dust and cumulative exposure, and for *F2RL3* CpG2 in association with respirable dust exposure.

Sensitivity analysis, including only welders with at least one measured data point for respirable dust (ie, without the welders with assessed exposure to respirable dust, N=183–186 depending on CpG site) or cumulative exposure, did not change the significance level or markedly change the effect estimates, and the results still showed a decrease in methylation with increasing exposure to welding fumes (supplementary table S5). In the sensitivity analysis of respirable dust among welders who had never smoked (N=123–125) (supplementary table S6), the association for *F2RL3* CpG1 became non-significant and the effect estimates changed (from β=-0.40, SE=0.16 to β=0.01, SE=0.2), whereas the effect estimates for *F2RL3* CpG2 remained similar, but became non-significant, to the main analysis (from β=-0.67 SE=0.23, P=0.004 to β=-0.59, SE=0.35, P=0.091). There was no association with cumulative exposure in welders who had never smoked (supplementary table S6).

*B3GNTL1* CpG1 showed significant positive associations with respirable dust (β=0.28, P=0.016) and cumulative exposure (β=0.02, P=0.011) (table 4). After adjustment for multiple testing, the association with cumulative exposure remained significant.

## Discussion

In this study, we investigated early lung cancer-related biomarkers in relation to occupational exposure to welding fumes. We found a significant decrease in DNA methylation of *B3GNTL1* CpG1 and CpG4 in welders compared with controls. A dose–response effect was observed in that *F2RL3* CpG1 and CpG2 methylation decreased with increasing personal respirable dust concentrations and cumulative exposure to welding fumes. In general, the effect estimate of the association with the selected biomarkers was subtle. However, since the respirable dust levels were below the limit of the current Swedish OEL (2.5 mg/m^3^), this study stresses that even exposure levels below the current OEL may be associated with epigenetic changes that could increase the risk of developing lung cancer.

*B3GNTL1* encodes a transmembrane protein, has 13 exons, and is on chromosome 17; it is expressed in several tissues, including in lung tissue. The B3GNTL1 protein is involved in the metabolism of proteins and *O*-linked glycosylation (45), but the mechanism linking *B3GNTL1* with cancer is unknown. Hypomethylation of *B3GNTL1* was observed in colorectal tumors in comparison to adjacent tissue (46). More recently, hypomethylation of *B3GNTL1*_cg13482620 (CpG6) was associated with an increased risk of lung cancer among non-smokers (37). Here, we observed hypomethylation of *B3GNTL1* CpG1 and CpG4 in welders compared with controls. These sites are close to one another, but most likely not within the regulatory region of *B3GNTL1*. The effect estimates of the two CpG sites increased when only including never-smokers, supporting the idea that a decrease in DNA methylation of *B3GNTL1* is not associated with smoking. We did not observe a dose–response relationship in the welding group in association with welding fume exposure, actually a positive effect estimate (hypermethylation) was observed with increasing exposure to respirable dust and cumulative exposure. The demethylation of specific sites of *B3GNTL1* may thus be linked to exposures we have not accounted for in our study cohort, which warrants further investigation.

In an earlier cross-sectional study at timepoint 1, we found hypomethylation of *F2RL3* CpG2 in the welders, in relation to exposure to respirable dust and among earlier smokers (36). In this study, we included a second timepoint and found that exposure to respirable dust was still associated with hypomethylation of *F2RL3* CpG2, and CpG1, in welders over time. Hypomethylation of *F2RL3* CpG1 and CpG2 was detected in our sensitivity analysis including only welders with measured respirable dust levels as well, which suggests that exposure to welding particles may play a role in the hypomethylation of this gene. It should be noted that we did not analyze CpG3, CpG4 and CpG5 in this study.

*F2RL3* is a protein-coding gene located on chromosome 19 and has two exons. It codes for protease-activated receptor-4, which is involved in the pathophysiology of neoplastic and cardiovascular disease (47). This protein takes part in blood coagulation, inflammation, and pain responses (48). Hypomethylation of *F2RL3*_cg03636183 (CpG2) was strongly correlated with tobacco smoking (33) as well as an increased risk of lung cancer (35). We also observed hypomethylation of *F2RL3* CpG1 and CpG2 in relation to smoking in our study. However, when excluding ever-smokers from our dose–response analysis in welders, we observed a decrease in the effect estimates and the *R^2^m* values, especially for CpG1 in association with respirable dust and cumulative exposure. When we excluded all welders who had ever smoked, the number of observations decreased from 220–223 to 125, which reduced the power of the analysis and thus could explain the drop in significance and effect estimates. The similar decrease in methylation in relation to smoking and respirable dust might suggest that similar mechanisms affect the methylation pattern of specific genes; however, it is more likely that residual confounding factors interfered with our main analysis, such as tobacco consumption earlier in life.

Two of the selected methylation sites, *AHRR* CpG3 and *F2RL3* CpG2, have been associated with tobacco smoking in previous studies (35, 49). In a recent case–control study (*N*=552 pairs) lower methylation of both *AHRR* CpG3 and *F2RL3* CpG2 in blood was associated with higher risk of lung cancer and the authors proposed that DNA methylation of the two studied CpG sites might be a predictor of future lung cancer (50). When comparing the selected biomarkers between never- and ever-smokers we also observed higher methylation of more than 4% for *AHRR* CpG1–3 and around 2% for *F2RL3* CpG1–2 in the never-smokers (supplementary table S3). Similar results were found when only including welders (supplementary table S7). No significant changes in the methylation of *AHRR* and *F2RL3* could be observed when comparing welders and controls; however, we observed a decrease in methylation of *F2RL3* in association with welding fume exposure, but to a lower extent than of smoking. The observed differences in methylation pattern, but not between exposure groups, could be due to the changes being related to smoking. Since our cohort consists of healthy workers and the welders are exposed to low amounts of particles, it is also possible that the follow-up time was not long enough to observe changes between exposure groups.

In previous cross-sectional studies based on study participants at timepoint 1, we found shorter TL in the welders (16) as well as an increase in mtDNAcn (17). These findings could not be confirmed in this study 6 years later (timepoint 2). One possible explanation is that even though we were able to detect differences between welders and controls at timepoint 1, these differences are no longer significant when including time in our statistical model. Differences at timepoint 1 might also be due to use of different materials or PPE, without influencing the respirable dust exposure. Another possible explanation might be differences in characteristics of the dropouts and new recruits; however, no significant differences between these two groups could be observed regarding mtDNAcn or TL (supplementary table S3).

A strength of this study is that the study groups consisted of non-smoking individuals at baseline. This is important because tobacco smoking is a major risk factor for lung cancer; therefore, we were able to study epigenetic changes in a population largely unaffected by an important co-exposure factor. Our study investigated lung cancer-related markers, but we measured the effect of welding on these biomarkers in blood and not lung tissue. Nevertheless, earlier studies have shown that blood-based biomarkers like the ones in the current study can predict lung cancer risk. Still the effect sizes were rather small and need to be interpreted with some caution. Another strength is that the exposure to welding fumes was assessed using multiple different measures. Still, there may be some misclassification of exposure since we were only able to measure respirable dust on one occasion during each timepoint, the calculation of the adjusted personal respirable dust exposure may introduce errors.

In conclusion, our study showed that welders had lower methylation of *B3GNTL1* CpG1 and CpG4 compared to controls as well as lower methylation of *F2RL3* CpG2 in association with respirable dust and cumulative exposure. Previous studies have associated both genes with future development of lung cancer. Components within the welding fumes likely play a role in the observed epigenetic modifications. The findings stress the need to further investigate the health effects of occupational welding exposure and, if needed, to adjust the current OEL accordingly.

## Supplementary material

Supplementary material

## References

[1] Guha N, Loomis D, Guyton KZ, Grosse Y, El Ghissassi F, Bouvard V, International Agency for Research on Cancer Monograph Working Group (2017). Carcinogenicity of welding, molybdenum trioxide, and indium tin oxide. Lancet Oncol.

[2] International Agency for Research on Cancer (IARC) Welding (2018). molybdenum trioxide, and indium tin oxide.

[3] (c2018). Statistic Sweden [Internet], The Swedish Occupational Register.

[4] Sjögren B (2013) Kunskapssammanställning. Hälsoeffekter av gaser och partiklar bildade vid svetsning [Knowledge compilation - Health effects from gases and particles emitted during welding].

[5] Arbetsmiljöverket (2018) Swedish Work Environment Authority. Occupational exposure limits. [Hygieniska gränsvärden].

[6] Sørensen AR, Thulstrup AM, Hansen J, Ramlau-Hansen CH, Meersohn A, Skytthe A (2007). Risk of lung cancer according to mild steel and stainless steel welding. Scand J Work Environ Health.

[7] Siew SS, Kauppinen T, Kyyrönen P, Heikkilä P, Pukkala E (2008). Exposure to iron and welding fumes and the risk of lung cancer. Scand J Work Environ Health.

[8] 't Mannetje A, Brennan P, Zaridze D, Szeszenia-Dabrowska N, Rudnai P, Lissowska J (2012). Welding and lung cancer in Central and Eastern Europe and the United Kingdom. Am J Epidemiol.

[9] Matrat M, Guida F, Mattei F, Cénée S, Cyr D, Févotte J, Icare Study Group (2016). Welding, a risk factor of lung cancer:the ICARE study. Occup Environ Med.

[10] Kim JY, Chen JC, Boyce PD, Christiani DC (2005). Exposure to welding fumes is associated with acute systemic inflammatory responses. Occup Environ Med.

[11] Shen S, Zhang R, Zhang J, Wei Y, Guo Y, Su L (2018). Welding fume exposure is associated with inflammation:a global metabolomics profiling study. Environ Health.

[12] Han SG, Kim Y, Kashon ML, Pack DL, Castranova V, Vallyathan V (2005). Correlates of oxidative stress and free-radical activity in serum from asymptomatic shipyard welders. Am J Respir Crit Care Med.

[13] Hoffmeyer F, Raulf-Heimsoth M, Lehnert M, Kendzia B, Bernard S, Berresheim H, Weldox Group (2012). Impact of different welding techniques on biological effect markers in exhaled breath condensate of 58 mild steel welders. J Toxicol Environ Health A.

[14] Marongiu A, Hasan O, Ali A, Bakhsh S, George B, Irfan N (2016). Are welders more at risk of respiratory infections?Findings from a cross-sectional survey and analysis of medical records in shipyard workers:the WELSHIP project. Thorax.

[15] Li H, Hedmer M, Kåredal M, Björk J, Stockfelt L, Tinnerberg H (2015). A Cross-Sectional Study of the Cardiovascular Effects of Welding Fumes. PLoS One.

[16] Li H, Hedmer M, Wojdacz T, Hossain MB, Lindh CH, Tinnerberg H (2015). Oxidative stress, telomere shortening, and DNA methylation in relation to low-to-moderate occupational exposure to welding fumes. Environ Mol Mutagen.

[17] Xu Y, Li H, Hedmer M, Hossain MB, Tinnerberg H, Broberg K (2017). Occupational exposure to particles and mitochondrial DNA - relevance for blood pressure. Environ Health.

[18] Gliga AR, Taj T, Hedmer M, Assarsson E, Rylander L, Albin M (2019). Mild steel welding is associated with alterations in circulating levels of cancer-related proteins. Arch Toxicol.

[19] Blackburn EH (1991). Structure and function of telomeres. Nature.

[20] Gisselsson D, Jonson T, Petersén A, Strömbeck B, Dal Cin P, Höglund M (2001). Telomere dysfunction triggers extensive DNA fragmentation and evolution of complex chromosome abnormalities in human malignant tumors. Proc Natl Acad Sci USA.

[21] Gilson E, Londoño-Vallejo A (2007). Telomere length profiles in humans:all ends are not equal. Cell Cycle.

[22] Wu X, Amos CI, Zhu Y, Zhao H, Grossman BH, Shay JW (2003). Telomere dysfunction:a potential cancer predisposition factor. J Natl Cancer Inst.

[23] Hosgood HD, Cawthon R, He X, Chanock S, Lan Q (2009). Genetic variation in telomere maintenance genes, telomere length, and lung cancer susceptibility. Lung Cancer.

[24] Sanchez-Espiridion B, Chen M, Chang JY, Lu C, Chang DW, Roth JA (2014). Telomere length in peripheral blood leukocytes and lung cancer risk:a large case-control study in Caucasians. Cancer Res.

[25] Doherty JA, Grieshober L, Houck JR, Barnett MJ, De Dieu Tapsoba J, Thornquist MD (2018). Nested case-control study of telomere length and lung cancer risk among heavy smokers in the β-Carotene and Retinol Efficacy Trial. Br J Cancer.

[26] Seow WJ, Cawthon RM, Purdue MP, Hu W, Gao YT, Huang WY (2014). Telomere length in white blood cell DNA and lung cancer:a pooled analysis of three prospective cohorts. Cancer Res.

[27] Lan Q, Cawthon R, Gao Y, Hu W, Hosgood HD, Barone-Adesi F (2013). Longer telomere length in peripheral white blood cells is associated with risk of lung cancer and the rs2736100 (CLPTM1L-TERT) polymorphism in a prospective cohort study among women in China. PLoS One.

[28] Shen M, Cawthon R, Rothman N, Weinstein SJ, Virtamo J, Hosgood HD (2011). A prospective study of telomere length measured by monochrome multiplex quantitative PCR and risk of lung cancer. Lung Cancer.

[29] Lee HC, Wei YH (2000). Mitochondrial role in life and death of the cell. J Biomed Sci.

[30] Hosgood HD, Liu CS, Rothman N, Weinstein SJ, Bonner MR, Shen M (2010). Mitochondrial DNA copy number and lung cancer risk in a prospective cohort study. Carcinogenesis.

[31] Wajed SA, Laird PW, DeMeester TR (2001). DNA methylation:an alternative pathway to cancer. Ann Surg.

[32] Gao X, Jia M, Zhang Y, Breitling LP, Brenner H (2015). DNA methylation changes of whole blood cells in response to active smoking exposure in adults:a systematic review of DNA methylation studies. Clin Epigenetics.

[33] Zhang Y, Yang R, Burwinkel B, Breitling LP, Brenner H (2014). F2RL3 methylation as a biomarker of current and lifetime smoking exposures. Environ Health Perspect.

[34] Zhang Y, Elgizouli M, Schöttker B, Holleczek B, Nieters A, Brenner H (2016). Smoking-associated DNA methylation markers predict lung cancer incidence. Clin Epigenetics.

[35] Fasanelli F, Baglietto L, Ponzi E, Guida F, Campanella G, Johansson M (2015). Hypomethylation of smoking-related genes is associated with future lung cancer in four prospective cohorts. Nat Commun.

[36] Hossain MB, Li H, Hedmer M, Tinnerberg H, Albin M, Broberg K (2015). Exposure to welding fumes is associated with hypomethylation of the F2RL3 gene:a cardiovascular disease marker. Occup Environ Med.

[37] Sandanger TM, Nøst TH, Guida F, Rylander C, Campanella G, Muller DC (2018). DNA methylation and associated gene expression in blood prior to lung cancer diagnosis in the Norwegian Women and Cancer cohort. Sci Rep.

[38] Hedmer M, Karlsson JE, Andersson U, Jacobsson H, Nielsen J, Tinnerberg H (2014). Exposure to respirable dust and manganese and prevalence of airways symptoms, among Swedish mild steel welders in the manufacturing industry. Int Arch Occup Environ Health.

[39] Gliga AR, Taj T, Wahlberg K, Lundh T, Assarsson E, Hedmer M (2020). Exposure to Mild Steel Welding and Changes in Serum Proteins With Putative Neurological Function-A Longitudinal Study. Front Public Health.

[40] Code of Federal Regulations [internet] (c2020). Method 201a—Determination of Pm10 and Pm2.5 Emissions From Stationary Sources (Constant Sampling Rate Procedure);Title 40 –Protection of Environment.

[41] Cawthon RM (2002). Telomere measurement by quantitative PCR. Nucleic Acids Res.

[42] Li H, Engström K, Vahter M, Broberg K (2012). Arsenic exposure through drinking water is associated with longer telomeres in peripheral blood. Chem Res Toxicol.

[43] Alhamdow A, Lindh C, Hagberg J, Graff P, Westberg H, Krais AM (2018). DNA methylation of the cancer-related genes F2RL3 and AHRR is associated with occupational exposure to polycyclic aromatic hydrocarbons. Carcinogenesis.

[44] R Core Team (2019) R:A language and environment for statistical computing.

[45] Zheng H, Li Y, Ji C, Li J, Zhang J, Yin G (2004). Characterization of a cDNA encoding a protein with limited similarity to β1, 3-N-acetylglucosaminyltransferase. Mol Biol Rep.

[46] Berman BP, Weisenberger DJ, Aman JF, Hinoue T, Ramjan Z, Liu Y (2011). Regions of focal DNA hypermethylation and long-range hypomethylation in colorectal cancer coincide with nuclear lamina-associated domains. Nat Genet.

[47] Zhang Y, Yang R, Burwinkel B, Breitling LP, Holleczek B, Schöttker B (2014). F2RL3 methylation in blood DNA is a strong predictor of mortality. Int J Epidemiol.

[48] Gomides LF, Duarte ID, Ferreira RG, Perez AC, Francischi JN, Klein A (2012). Proteinase-activated receptor-4 plays a major role in the recruitment of neutrophils induced by trypsin or carrageenan during pleurisy in mice. Pharmacology.

[49] Shenker NS, Ueland PM, Polidoro S, van Veldhoven K, Ricceri F, Brown R (2013). DNA methylation as a long-term biomarker of exposure to tobacco smoke. Epidemiology.

[50] Baglietto L, Ponzi E, Haycock P, Hodge A, Bianca Assumma M, Jung CH (2017). DNA methylation changes measured in pre-diagnostic peripheral blood samples are associated with smoking and lung cancer risk. Int J Cancer.

