# Supplementary material for: Cancer-related changes and low-to-moderate exposure to welding fumes: A longitudinal study
Source: Scand J Work Environ Health. 2021 Dec 30;48(1):21–30. doi: 10.5271/sjweh.3988 (PMC8729166; doi:10.5271/sjweh.3988)
Supplement: Supplementary material [file SJWEH-48-21-S001.pdf]

Cancer-related changes and low-to-moderate exposure to welding fumes: A longitudinal study[1]

by Ulrike Maria Dauter, MSc Ayman Alhamdow, PhD, Andrea Cediel-Ulloa, MSc, Anda Roxana Gliga, PhD, Maria Albin, PhD, Karin Broberg, PhD

1. *Supplementary material*
2. *Correspondence to: Karin Broberg, Professor, Institute of Environmental Medicine, Karolinska Institutet, Nobels väg 13, 171 77, Stockholm, Sweden. [E-mail: karin.broberg@ki.se]*

Relative TL was calculated using the ratio of the amount of telomere repeats (T) to the amount of a single-copy gene (S), i.e. *HBB* (hemoglobin beta chain). A standard curve for qPCR was created by using serially diluted DNA (16, 8, 4, 2, 1 and 0.5 ng/µL) and two independent qPCR runs were conducted, one for the telomere repeats and one for *HBB*. All samples and negative controls were run in triplicate. The coefficient of determination (R^2) was >0.99 for all telomere and *HBB* runs. A standard deviation of 0.2 for Ct was accepted.

Two independent qPCR runs were performed: one to measure the mtDNAcn and one to measure *HBB*. For the mtDNAcn assay, 7.5 µL of KAPA master mix (KAPA SYBR FAST Master Mix 2×, Optimized for LightCycler 480; Kapa Biosystems, Wilmington, US) was mixed with a 2.5-µL DNA sample. Samples, the standard curve, and the negative control were run in triplicate. Similar to the TL assay, >0.99 was used as a coefficient of determination and a standard deviation of the Ct-value of 0.2 was accepted for triplicates.

The DNA samples (500 ng) extracted from peripheral blood were randomized in 96-well plates and bisulfite treated using EZ-96 DNA Methylation-Gold Kit (Zymo Research, Irvine, CA). The DNA was amplified for the target regions using a standard thermal cycler (SimpliAmp, Thermo Fisher Scientific, Carlsbad, CA); thereafter, randomly selected samples from each PCR plate were run on an agarose gel to ensure specificity of the PCR primer pairs. PCR conditions for all assays are described in Supplementary Table 1.

The percentage of DNA methylation of each CpG site was measured using the pyrosequencing platform PyroMark Q96 ID (Qiagen). All PCR and pyrosequencing reagents were purchased from Qiagen, except Streptavidin Sepharose High Performance (GE Healthcare, Uppsala, Sweden). Methylated and unmethylated human DNA controls (bisulfite-treated; Qiagen) were included in each PCR and pyrosequencing run along with negative controls. A bisulfite-treated calibrator DNA sample was run in duplicate in each plate to calculate intra- and inter-plate coefficients of variation (%CV), which ranged between 0 and <5.0% for the intra-plate %CV and between 1.0 and <4.0% for the inter-plate %CV for all CpG sites of all assays.

Statistical Analysis

Differences in the methylation of the selected CpG sites, mtDNAcn, TL, and the exposure variables between people who have smoked at some point in their lives (ever-smokers) and those who have never smoked (never-smokers) were assessed using the Wilcoxon test. Differences between dropouts and the remaining individuals of timepoint 1, dropouts and new recruits, and new recruits and the remaining individuals of timepoint 2 were assessed using the Kruskal-Wallis rank sum test, followed by Dunn's post hoc if applicable, when comparing three or more groups, and the Fisher's exact test for categorial variables.

The relationship between smoking status and the outcome and exposure variables was assessed with the Kruskal-Wallis test, followed by Dunn's post hoc test. The relationship between ever-smokers and never-smokers against outcome and exposure variables was assessed using the Wilcoxon test.

Supplementary Table S1: Detailed information about pyrosequencing assays for *F2RL3, AHRR*, and *B3GNTL1* (obtained 14 February 2020)

F2RL3[c]		
CpG site No.	CpG1	CpG2
Position[a]	Chr19: NC_000019.10; 16889785	Chr19: NC_000019.10; 16889774
Forward primer 5'-	GTGGGGTTGTAGGTTAATGGGT	
Reverse primer 5'-	[Biotin]-CCAACAACAACACTAAACCATAC ATAT	
Sequencing primer	GTTGGAGTTGTGGGTG	
Sequence analyzed[b]	TTGGTTA**Y***GTAGGTATTT**Y**GGTTGTTTT TTATTATGTTGT TGATG	
PCR conditions	95°C for 15 min, 45 cycles of: 94°C for 30 s, 52°C for 30 s, 72°C for 30 s; 72°C for 10 min	
Amplicon size	235 nucleotides	

AHRR[c]			
CpG site No	CpG1	CpG2	CpG3
Position[a]	Chr5: NC_000005.10; 373238	Chr5: NC_000005.10; 373240	Chr5: NC_000005.10; 373263
Forward primer 5'-	ATAGGGGTTGTTTAGGTTATAGATT		
Reverse primer 5'-	[Biotin]-ACCTATCCCCTACCTCCC		
Sequencing primer	ATTGTTTATTTTTGAGAGGGTA		
Sequence analyzed[b]	GTTTTG TTT**Y**G**Y**GG TTTTGG TTTTGTTTTG TATT**Y**[#]GGTTGGG TTTTATTTGATA		
PCR conditions	95°C for 15 min, 45 cycles of: 94°C for 30 s, 57°C for 30 s, 72°C for 30 s; 72°C for 10 min		
Amplicon size	246 nucleotides		

B3GNTL1[c]						
CpG site No	CpG1	CpG2	CpG3	CpG4	CpG5	CpG6
Position[a]	Chr17: NC_000017.11; 83015699	Chr17: NC_000017.11; 83015694	Chr17: NC_000017.11; 83015690	Chr17: NC_000017.11; 83015686	Chr17: NC_000017.11; 83015682	Chr17: NC_000017.11; 83015679
Forward primer 5'-	TAGTAGTTGTTTTGATAGGAGGGGTAGT					
Reverse primer 5'-	ACACACAATATATACCAAAATCCCAATTC					
Sequence primer	GAGGGGTAGTTTAGG					
Sequence analyzed[b]	T**Y**GTGA**Y**GTT**Y**GTT**Y**GGT**Y**GT**Y**[§]GTTTATTTTTGTTGTGTGGTTTGGTT					
PCR conditions	95°C for 15 min, 45 cycles of: 94°C for 30 s, 57°C for 30 s, 72°C for 30 s; 72°C for 10 min					
Amplicon size	139 nucleotides					

[a]According to the Genome Reference Consortium Human Build 38 patch release 7 (GRCh38.p7). [b]Y is either C or T. [c]The volume of PCR product used for pyrosequencing was 20 µL. *Corresponds to cg03636183. [#]Corresponds to cg05575921. [$]Corresponds to cg13482620.

Supplementary Table S2: CpG methylation sites of selected genes in ever- and never-smokers of the selected methylation sites as well as the mtDNA copy number and telomere length for both groups, including the mean value difference and the results of the Wilcoxon rank sum test when comparing the two groups with one another.

		Ever-smokers (*n*=205)					Never-smokers (*n*=300)						
		Mean	Median	5%	95%	St. dev	Mean	Median	5%	95%	St. dev	Difference mean (never - ever)	*p* value
AHRR	CpG 1	71.07	73.29	51.02	82.24	10.49	75.67	76.14	65.56	84.21	5.98	4.60	<0.001
	CpG 2	63.20	65.06	45.53	73.19	8.98	68.03	68.92	58.83	74.35	4.75	4.83	<0.001
	CpG3	82.86	85.38	62.33	92.97	10.17	88.50	88.97	80.74	95.21	4.39	5.64	<0.001
F2RL3	CpG 1	70.44	70.87	64.54	74.94	3.82	72.43	72.49	68.86	76.05	2.22	1.99	<0.001
	CpG 2	91.22	91.39	82.77	99.97	5.30	93.31	93.12	87.33	100.00	3.90	2.08	<0.001
B3GNTL1	CpG 1	98.07	98.59	93.87	100.00	2.14	97.67	98.33	93.22	100.00	3.52	-0.41	0.156
	CpG 2	96.93	96.94	95.84	97.82	0.70	96.89	96.91	95.64	97.76	0.77	-0.04	0.370
	CpG 3	95.62	95.78	90.84	100.00	2.76	95.69	95.70	90.63	100.00	2.87	0.06	0.810
	CpG 4	96.11	96.05	91.60	100.00	2.58	96.30	96.46	91.70	100.00	2.83	0.19	0.275
	CpG 5	98.13	98.35	95.26	100.00	1.56	97.94	98.06	94.91	100.00	1.88	-0.19	0.251
	CpG 6	96.03	95.92	92.96	99.54	2.06	95.88	96.00	91.71	99.33	2.23	-0.15	0.717
mtDNA*		1.11	1.07	0.77	1.55	0.24	1.16	1.11	0.81	1.64	0.27	0.05	0.049
Telomere length		0.93	0.93	0.68	1.22	0.17	0.96	0.94	0.69	1.26	0.18	0.03	0.147

* mitochondrial DNA copy number

Supplementary Table S3: Differences between individuals from timepoint 1 (cycle 1) and dropouts, individuals from timepoint 2 (cycle 2) and new recruits as well as between dropouts from timepoint 1 and new recruits from timepoint 2. Wilcoxon Unpaired Two-Sample test was used for calculations of continuous variables, Fisher's exact test for categorial variables, Kruskal-Wallis test for categorical variables with 3+ categories.

	Cycle 1 *vs* Dropouts	Cycle 2 *vs* new recruits	Dropouts *vs* new recruits
	p value	*p* value	*p* value
Education (university or higher)	0.913	0.973	0.858
Residence (large and small cities)	0.052	0.141	0.175
Hobby exposure to particles	0.254	0.764	0.320
Smoking history (ever smoked)	0.264	0.615	0.735
Current snus use	1	0.036	0.147
Alcohol intake (3+ times/week)	0.889	0.170	0.591
Vegetable intake (5+ times/week)	0.546	0.831	0.463
Physical activity (moderate/high)	0.321	0.796	0.184
Cancer history	0.430	0.335	0.307
Family cancer history	0.215	0.893	0.112

Supplementary Table S4: Association of epigenetic marker in welders and controls using linear mixed modelling for analysis comparing welders and controls.

		R^2_m (%)	*β* (SE)	*p* value	*n*[a]
**Never-welding controls (adj. age, BMI, smoking (ever))**					
AHRR	CpG1	2	-0.99 (0.95)	0.297	446
	CpG2	1	-0.70 (0.79)	0.375	446
	CpG3	4	-0.65 (0.81)	0.421	446
F2RL3	CpG1	6	-0.15 (0.31)	0.640	446
	CpG2	1	0.33 (0.43)	0.444	446
B3GNTL1	CpG1	2	-0.54 (0.30)	0.076	445
	CpG2	3	0.04 (0.07)	0.572	445
	CpG3	0	0.09 (0.27)	0.729	445
	CpG4	1	-0.66 (0.26)	0.011	445
	CpG5	2	0.42 (0.17)	0.012	443
	CpG6	1	0.04 (0.21)	0.842	443
mtDNA*		1	-0.04 (0.03)	0.171	446
Telomere length		5	0.02 (0.02)	0.226	446
**Never-smokers (adj. age, BMI)**					
AHRR	CpG1	0	-0.41 (0.84)	0.627	300
	CpG2	1	-0.33 (0.67)	0.624	300
	CpG3	0	0.00 (0.58)	1.000	300
F2RL3	CpG1	2	0.13 (0.26)	0.617	300
	CpG2	2	0.82 (0.45)	0.068	300
B3GNTL1	CpG1	3	-1.00 (0.41)	0.015	299
	CpG2	4	-0.04 (0.09)	0.683	299
	CpG3	0	0.05 (0.33)	0.870	299
	CpG4	2	-0.69 (0.33)	0.035	299
	CpG5	2	0.56 (0.23)	0.014	297
	CpG6	1	-0.15 (0.26)	0.560	297
mtDNA*		1	-0.02 (0.03)	0.530	300
Telomere length		1	0.02 (0.02)	0.473	300

[a] number of observations, * mitochondrial DNA copy number

Supplementary Table S5: Association of the epigenetic markers in welders with the exposure variable expressed as respirable dust (adjusted for use of personal protective equipment) and cumulative exposure using linear mixed modelling for analysis. Only welders with at least one measured datapoint for respirable dust were included. The models were adjusted for age, BMI, and smoking (ever-smoking).

		R^2_m (%)	β (SE)	*p* value	*n*[a]
**Respirable dust**					
AHRR	CpG1	2	0.29 (0.37)	0.427	186
	CpG2	4	-0.22 (0.29)	0.436	186
	CpG3	11	-0.20 (0.34)	0.561	186
F2RL3	CpG1	10	-0.42 (0.16)	0.008	186
	CpG2	6	-0.68 (0.24)	0.004	186
B3GNTL1	CpG1	6	0.27 (0.13)	0.032	186
	CpG2	2	0.04 (0.13)	0.359	186
	CpG3	0	-0.01 (0.16)	0.968	186
	CpG4	2	0.11 (0.16)	0.475	186
	CpG5	1	-0.04 (0.09)	0.679	184
	CpG6	3	-0.02 (0.12)	0.872	184
mtDNA*		0	0.00 (0.01)	0.774	185
Telomere length		4	-0.01 (0.01)	0.415	185
**Cumulative exposure**					
AHRR	CpG1	3	-0.04 (0.03)	0.162	185
	CpG2	5	-0.04 (0.02)	0.134	185
	CpG3	11	-0.04 (0.02)	0.124	185
F2RL3	CpG1	15	-0.04 (0.01)	< 0.001	185
	CpG2	4	-0.04 (0.02)	0.026	185
B3GNTL1	CpG1	6	0.02 (0.01)	0.02	185
	CpG2	2	0.00 (0.00)	0.473	185
	CpG3	0	0.00 (0.01)	0.837	185
	CpG4	2	0.01 (0.01)	0.379	185
	CpG5	1	0.00 (0.01)	0.552	183
	CpG6	3	0.00 (0.01)	0.878	183
mtDNA*		0	0.00 (0.00)	0.664	184
Telomere length		3	0.00 (0.00)	0.885	184

[a] number of observations, * mitochondrial DNA copy number

Supplementary Table S6: Association of the epigenetic markers in welders with the exposure variable expressed as respirable dust (adjusted for use of personal protective equipment), cumulative exposure, and welding years using linear mixed modelling for analysis. Only never-smoking welders were included. The models were adjusted for age and BMI.

		R^2_m (%)	*β* (SE)	*p* value	*n*[a]
**Respirable dust**					
AHRR	CpG1	0	-0.09 (0.47)	0.857	125
	CpG2	2	-0.30 (0.37)	0.416	125
	CpG3	0	0.07 (0.43)	0.876	125
F2RL3	CpG1	1	0.01 (0.20)	0.961	125
	CpG2	6	-0.59 (0.35)	0.091	125
B3GNTL1	CpG1	9	0.49 (0.21)	0.017	125
	CpG2	13	0.07 (0.07)	0.290	125
	CpG3	1	-0.18 (0.26)	0.487	125
	CpG4	1	0.18 (0.27)	0.498	125
	CpG5	1	-0.06 (0.16)	0.692	123
	CpG6	2	0.06 (0.21)	0.773	123
mtDNA*		1	0.00 (0.02)	0.985	125
Telomere length		2	-0.02 (0.02)	0.206	125
**Cumulative exposure**					
AHRR	CpG1	1	-0.03 (0.05)	0.570	125
	CpG2	2	0.00 (0.03)	0.890	125
	CpG3	1	0.01 (0.03)	0.688	125
F2RL3	CpG1	2	-0.02 (0.01)	0.247	125
	CpG2	4	0.00 (0.03)	0.877	125
B3GNTL1	CpG1	7	0.02 (0.02)	0.130	125
	CpG2	12	0.00 (0.01)	0.616	125
	CpG3	2	-0.03 (0.02)	0.145	125
	CpG4	1	0.01 (0.02)	0.591	125
	CpG5	2	0.01 (0.01)	0.342	123
	CpG6	2	-0.01 (0.02)	0.743	123
mtDNA*		1	0.00 (0.00)	0.748	125
Telomere length		1	0.00 (0.00)	0.876	125
**Welding years**					
AHRR	CpG1	2	0.11 (0.08)	0.177	135
	CpG2	2	0.07 (0.06)	0.230	135
	CpG3	4	0.11 (0.06)	0.056	135
F2RL3	CpG1	3	-0.03 (0.03)	0.288	135
	CpG2	3	-0.02 (0.05)	0.685	135
B3GNTL1	CpG1	4	0.07 (0.05)	0.215	135
	CpG2	11	0.00 (0.01)	0.620	135
	CpG3	1	-0.04 (0.03)	0.296	135
	CpG4	1	-0.02 (0.03)	0.621	135
	CpG5	3	0.04 (0.02)	0.079	133
	CpG6	6	-0.06 (0.03)	0.021	133
mtDNA*		2	0.00 (0.00)	0.443	135
Telomere length		1	0.00 (0.00)	0.567	135

[a] number of observations, * mitochondrial DNA copy number

Supplementary Table S7: CpG methylation sites of selected genes in welders and controls of the selected methylation sites as well as the mtDNA copy number and telomere length for both groups, including the mean value difference and the results of the Wilcoxon rank sum test when comparing the two groups. The lower part of the table shows the differences between ever- and never-smoking welders.

		Welders (*n*=246)		Controls (*n*=261)			
		Mean	St. dev	Mean	St. dev	Difference mean (controls - welders)	*p* value
AHRR	CpG 1	73.57	8.82	74.06	8.00	0.49	0.434
	CpG 2	65.86	7.33	66.28	7.03	0.42	0.423
	CpG3	86.08	8.09	86.38	7.50	0.30	0.625
F2RL3	CpG 1	71.60	3.17	71.63	3.09	0.03	0.927
	CpG 2	92.84	4.43	92.11	4.78	-0.73	0.138
B3GNTL1	CpG 1	97.51	3.75	98.14	2.11	0.63	0.008
	CpG 2	96.91	0.71	96.90	0.77	-0.01	0.597
	CpG 3	95.67	2.75	95.64	2.89	-0.03	0.800
	CpG 4	95.99	2.75	96.46	2.69	0.47	0.043
	CpG 5	98.26	1.62	97.84	1.86	-0.42	0.006
	CpG 6	95.94	2.25	95.91	2.09	-0.03	0.712
mtDNA*		1.12	0.25	1.15	0.27	0.03	0.268
Telomere length		0.95	0.18	0.94	0.17	-0.01	0.636
**Welders divided into ever- and never-smokers**							
		Ever-smokers (*n*=110)		Never-smokers (*n*=135)			
		Mean	St. dev	Mean	St. dev	Difference mean (never - ever)	*p* value*
AHRR	CpG 1	71.11	10.78	75.45	6.26	4.34	0.006
	CpG 2	63.36	9.02	67.85	4.78	4.49	0.000
	CpG3	83.07	10.12	88.50	4.79	5.43	0.000
F2RL3	CpG 1	70.55	3.81	72.47	2.19	1.92	0.000
	CpG 2	91.69	4.73	93.77	3.96	2.08	0.001
B3GNTL1	CpG 1	98.02	1.97	97.09	4.70	-0.93	0.047
	CpG 2	96.97	0.58	96.87	0.80	-0.10	0.102
	CpG 3	95.65	2.72	95.72	2.79	0.07	0.841
	CpG 4	96.05	2.54	95.93	2.91	-0.12	0.983
	CpG 5	98.27	1.51	98.25	1.72	-0.02	0.779
	CpG 6	96.17	2.09	95.79	2.36	-0.38	0.258
mtDNA*		1.09	0.22	1.15	0.27	0.06	0.175
Telomere length		0.94	0.18	0.97	0.18	0.03	0.188
Cumulative exposure		14.21	25.80	11.01	13.87	-3.20	0.670
Respirable dust		2.14	2.03	1.61	1.67	-0.53	0.017
Respirable dust adjusted		1.25	1.53	0.98	0.99	-0.27	0.291
Welding years		11.50	8.14	11.96	8.62	0.46	0.855

* mitochondrial DNA copy number

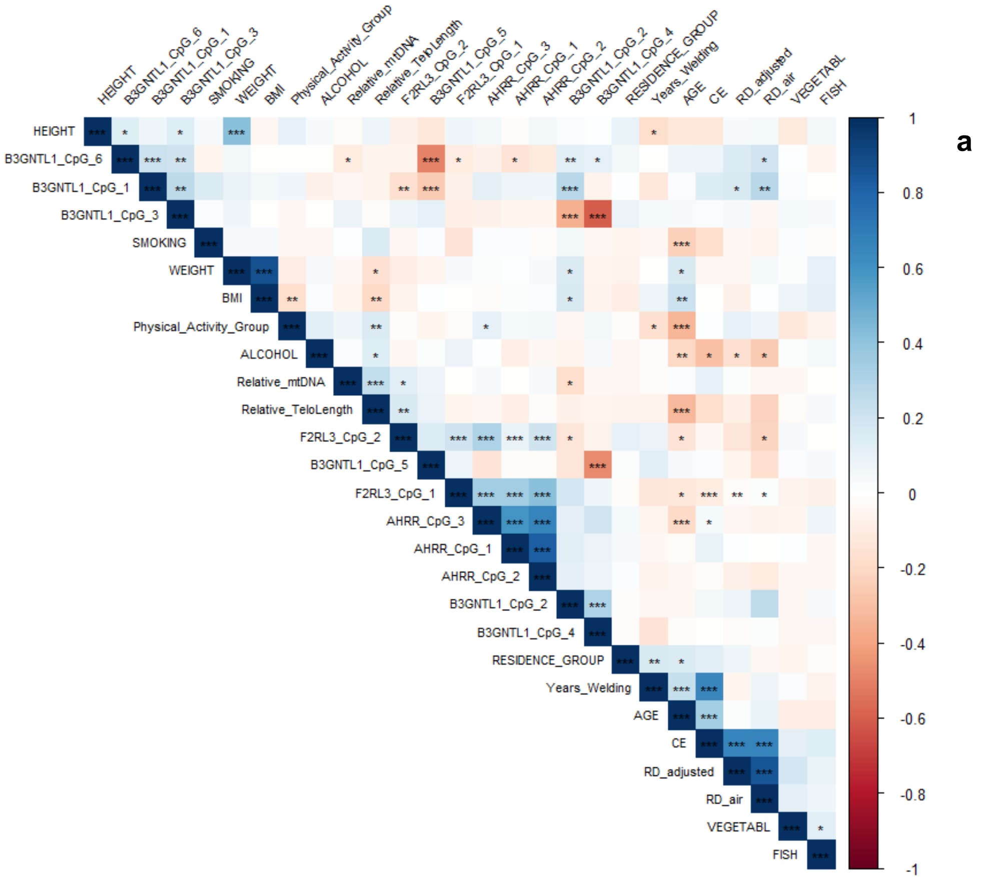

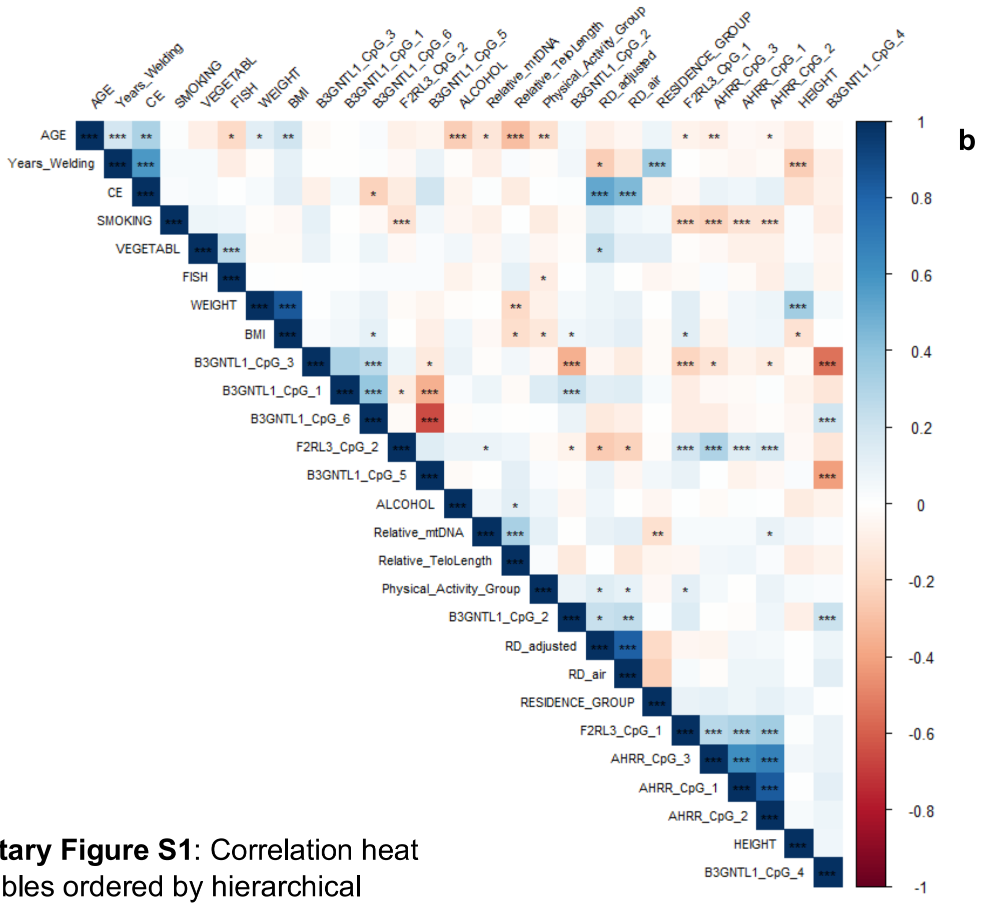

Supplementary Figure S1: Correlation heat map of variables ordered by hierarchical clustering, **a** – timepoint 1; **b** – timepoint 2
